# Immunological Tolerance to Muscle Autoantigens Involves Peripheral Deletion of Autoreactive CD8^+^ T Cells

**DOI:** 10.1371/journal.pone.0036444

**Published:** 2012-05-03

**Authors:** Emilie Franck, Carole Bonneau, Laetitia Jean, Jean-Paul Henry, Yann Lacoume, Anna Salvetti, Olivier Boyer, Sahil Adriouch

**Affiliations:** 1 Inserm, U905, Rouen, France; 2 University of Rouen, Institute for Research and Innovation in Biomedicine (IRIB), Normandy, France; 3 Inserm, U1096, Rouen, France; 4 Inserm, U758, Ecole Normale Supérieure de Lyon (ENS), Lyon, France; 5 Department of Immunology, Rouen University Hospital, Rouen, France; Saint Louis University School of Medicine, United States of America

## Abstract

Muscle potentially represents the most abundant source of autoantigens of the body and can be targeted by a variety of severe autoimmune diseases. Yet, the mechanisms of immunological tolerance toward muscle autoantigens remain mostly unknown. We investigated this issue in transgenic SM-Ova mice that express an ovalbumin (Ova) neo-autoantigen specifically in skeletal muscle. We previously reported that antigen specific CD4^+^ T cell are immunologically ignorant to endogenous Ova in this model but can be stimulated upon immunization. In contrast, Ova-specific CD8^+^ T cells were suspected to be either unresponsive to Ova challenge or functionally defective. We now extend our investigations on the mechanisms governing CD8^+^ tolerance in SM-Ova mice. We show herein that Ova-specific CD8^+^ T cells are not detected upon challenge with strongly immunogenic Ova vaccines even after depletion of regulatory T cells. Ova-specific CD8^+^ T cells from OT-I mice adoptively transferred to SM-Ova mice started to proliferate *in vivo*, acquired CD69 and PD-1 but subsequently down-regulated Bcl-2 and disappeared from the periphery, suggesting a mechanism of peripheral deletion. Peripheral deletion of endogenous Ova-specific cells was formally demonstrated in chimeric SM-Ova mice engrafted with bone marrow cells containing T cell precursors from OT-I TCR-transgenic mice. Thus, the present findings demonstrate that immunological tolerance to muscle autoantigens involves peripheral deletion of autoreactive CD8^+^ T cells.

## Introduction

Skeletal muscle is an abundant tissue constituting up to 40% of the total weight and, consequently, represents the most prominent source of potential autoantigens in the body. Yet, the precise mechanisms governing immune tolerance toward muscle antigens are still poorly understood. In the field of vaccination, muscle is considered as an immunogenic site and intramuscular injections have been used to elicit efficient immune priming even with DNA vaccines. This may be partly related to the intrinsic capacity of myoblasts and myocytes to act as facultative antigen presenting cells (APC) during local inflammation [1]. One particular immunological feature of muscle cells is that they do not express detectable levels of MHC-I molecules in physiological conditions while they can up-regulate the expression of both MHC-I and MHC-II after myoinjury or upon inflammation [2]–[5]. Local inflammation drives myoblast conversion into non-professional APC expressing not only MHC molecules but also some B7-related co-stimulatory/regulatory molecules. For instance, ICOS-L, BB-1 and PD-L1 have been detected on muscle cells cultured in *vitro* and on muscle fibers of patients with myositis [6]–[8]. Muscle cells are also equipped with multiple Toll-like receptors and have the capacity to secrete chemokines and pro-inflammatory cytokines such as IL-1α, IL-1β, TNF-α, IL-6, IL-8, IL-15, RANTES or MCP-1 [8]. Monocytes and others leukocytes are actively recruited in inflamed muscles where they participate to the local immune response and contribute to muscle regeneration at later time points [9]. Hence, the muscle tissue expresses multiple immunologically relevant molecules that actively support immune responses directed toward antigens present in muscle.

Despite the apparent immunogenicity of the muscle site, autoimmune diseases targeting skeletal muscle remain relatively rare. Indeed, polymyositis, the archetype of autoimmune muscle disease, has a reported annual incidence ranging, depending on the diagnostic criteria, between 1 and 8 cases per million [10]–[12]. This suggests that the muscle tissue may be particularly resistant to autoimmunity. Polymyositis is characterized histologically by muscle infiltration of CD8^+^ T cells and macrophages, and by the expression of MHC-I at the surface of myofibers even at distance from the cellular infiltrate [10]–[12]. Interestingly, activated CD8^+^ T cells displaying an oligoclonal TCR repertoire have been detected both in infiltrated muscles as well as in the circulation of patients with polymyositis, indicating that the immune attack is antigen-driven and directed against as yet unidentified autoantigens [13], [14]. Even if the pathophysiological conditions leading to immune activation in polymyositis are still not fully understood, over-expression of MHC molecules by muscle fibers and local secretion of pro-inflammatory cytokines probably contribute to the breakage of immune tolerance against muscle autoantigens.

To study the mechanisms leading to tolerance toward muscle-expressed autoantigens, we previously generated a transgenic (Tg) model in which mice express a membrane-bound form of ovalbumin (Ova) exclusively in the skeletal muscle [15]. In this model, named SM-Ova, the Ova-transgene has been placed under the control of a mutated version of the muscle creatine kinase (MCK) promoter allowing its expression in the skeletal muscle but not in the myocardium. Immunological analyses of SM-Ova mice after breeding with Tg mice expressing an MHC-I restricted (OT-I) or MHC-II restricted (OT-II) Ova-specific TCR revealed an absence of thymic deletion of Ova-specific clones and an absence of obvious signs of autoimmunity in unmanipulated mice. In single Tg SM-Ova mice, Ova-specific CD4^+^ T cell dependant responses as well as anti-Ova IgG antibody production were readily detected after immunization indicating that CD4^+^ T cells are not tolerant but rather ignorant of muscle-expressed autoantigens [15]. In striking contrast, specific cytotoxic activities against Ova-pulsed targets could not be detected after immunization of SM-Ova suggesting that Ova-specific CD8^+^ T cells were either unresponsive to Ova challenge, functionally defective or had been deleted from the lymphocyte repertoire. Here, we explored this issue and demonstrate that Ova-reactive CD8^+^ T cells are not ignorant nor blocked in their cytotoxic activity but are rather selectively deleted from the periphery of SM-Ova mice. Hence, the mechanisms preventing activation of T cells reactive to muscle-expressed autoantigens appear to be fundamentally different for CD4^+^ T cells that ignore muscle antigens and for CD8^+^ T cells that are physically deleted from the peripheral repertoire.

## Results

### Tolerance of the CD8^+^ compartment in SM-Ova mice

**Figure 1:**
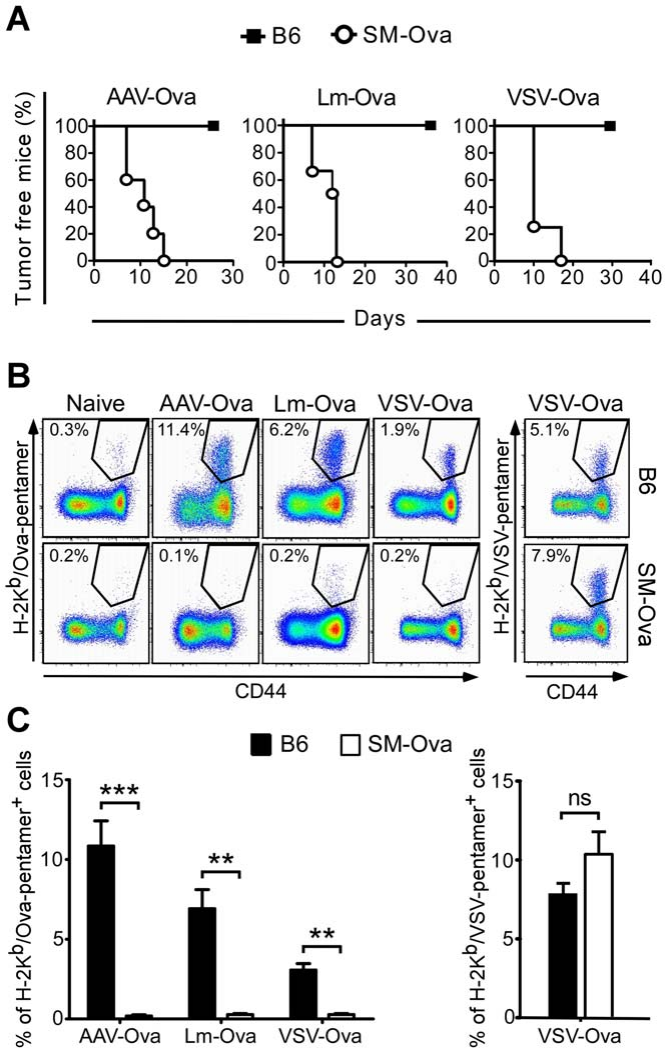
Lack of detectable Ova-specific CD8^+^ T cell responses in immunized SM-Ova mice. (**A**) B6 or SM-Ova mice were immunized with rAAV-Ova, replicative VSV-Ova or live Lm-Ova. Seven days after infection with VSV-Ova or Lm-Ova, or 14 days after injection of rAAV-Ova, mice were inoculated with 1×10^6^ Ova-bearing EG-7 tumor cells and monitored during 30–40 days for tumor development. (**B**) In other groups of mice, animals were immunized with the same vaccines and splenocytes were analyzed 7 or 14 days after by flow cytometry to evaluate the percentage of CD8^+^ T cell recognizing the immunodominant peptides of Ova or VSV-associated nucleoprotein using H-2K^b^/Ova_257–264_ or H-2K^b^/VSV pentamers staining, respectively. Representative flow cytometry profiles are shown and numbers indicate percentage of pentamer-positive cells in the CD8^+^-gated population. Background staining using H-2K^b^/VSV pentamers were always below 0.3% of CD8^+^ cells as also shown for the staining using H-2K^b^/Ova_257–264_ pentamers (**C**) Bar graphs represent mean percentages of CD8^+^ T cells positively stained with the indicated H-2K^b^/Ova_257–264_ or H-2K^b^/VSV pentamers in B6 (black bars) or SM-Ova (open bars). Data are representative of 3 independent experiments, each one performed with 5–7 mice per group.

The capacity of SM-Ova mice to mount a cytotoxic immune response was reevaluated *in vivo* using vaccines known to induce strong CTL responses. For that, B6 or SM-Ova mice were challenged with Ova-encoding vaccines that consisted of a defective adeno-associated viral vector rAAV-Ova [16], a replicative VSV-Ova virus [17] or a live Lm-Ova bacterial strain [18]. They were then evaluated for their capacity to reject an Ova-bearing EG7 tumor. While unimmunized B6 mice developed measurable tumors 5–10 days after EG7 inoculation (not shown), all B6 mice immunized with Ova-vaccines remained tumor-free throughout the entire evaluation period (Fig. 1A). This illustrates the sensitivity of this tumor model to Ova-specific immune responses as previously shown [19]. In contrast, SM-Ova mice were unable to reject Ova-expressing EG-7 tumor cells in the same experimental conditions, indicating that they could not generate an Ova-specific cytotoxic response *in vivo* (Fig. 1A). Since we already reported that SM-Ova mice generate normal Ova-specific CD4^+^ T cell and B cell responses after immunization [15], we focused our evaluations on the priming of Ova-specific CD8^+^ cells. Mice were immunized with Ova-vaccines and euthanized 7 or 14 days after to assess the percentage of CD8^+^ T cells recognizing the MHC-I restricted immunodominant Ova-derived peptide using H-2K^b^/Ova_257–264_ pentamer staining. Results showed a significant expansion of Ova-specific CD8^+^ splenocytes in B6 mice after immunization with VSV-Ova, Lm-Ova or rAAV-Ova vaccines (Fig. 1B and C). In striking contrast, SM-Ova mice immunized in the same condition did not display any detectable Ova-specific CD8^+^ T cell response above the background level (Fig. 1B and C). This was not due to a global defect in CD8^+^ T cells priming since SM-Ova mice immunized with VSV-Ova displayed a normal CD8^+^ response against the VSV nucleoprotein (Fig. 1B and C). We even noticed in SM-Ova mice an increase in the frequencies of CD8^+^ cells directed against the VSV nucleoprotein which could possibly reflect a better immune response to this antigen in the absence of Ova-specific CD8^+^ T response (Fig. 1B and C). Collectively these data show that SM-Ova mice are selectively defective in their capacity to mount a CD8^+^ T cells response against Ova-derived antigens. Thus, the absence of cytotoxic activities in SM-Ova mice after an Ova challenge was not secondary to a functional defect but was rather due to the absence of Ova-specific CD8^+^ T cells or to their incapacity to expand in vivo.

### Treg do not play a major inhibitory role on the Ova-specific CD8^+^ T cells response in SM-Ova mice

**Figure 2:**
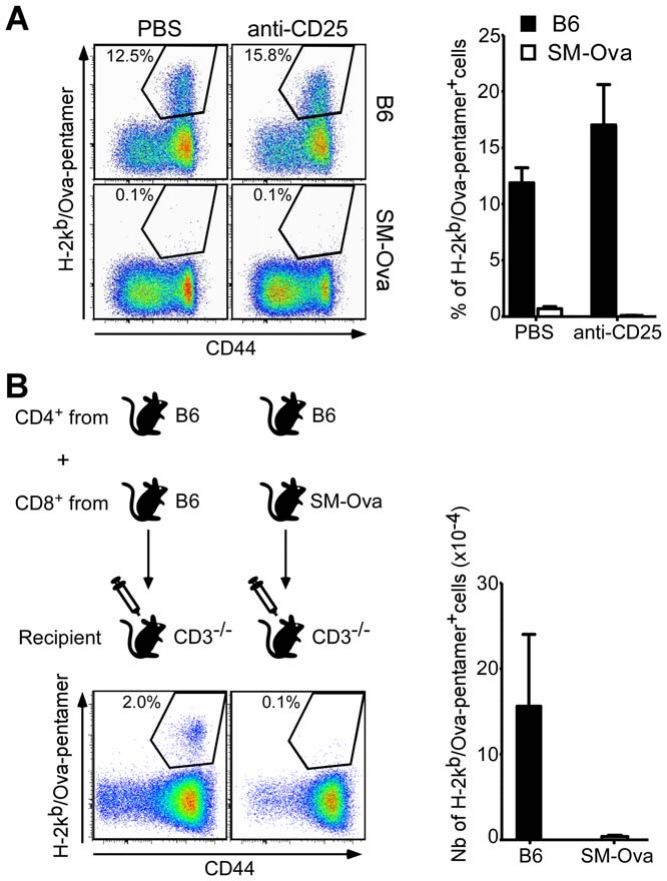
Treg depletion or adoptive cell transfer to CD3^−/−^ mice is not sufficient to restore an Ova-specific CD8^+^ T cell response. (**A**) B6 (n = 3) and SM-Ova (n = 3) mice were injected i.p. at day −4 and day −1 with 250 µg of anti-CD25 (PC-61) antibody to inactivate endogenous Treg, or with PBS, followed by the injection of rAAV-Ova at day 0. Splenocytes from treated mice were collected 14 days after immunization and analyzed as before by flow cytometry for the presence of Ova-specific CD8^+^ cells using H-2K^b^/Ova_257–264_ pentamer staining. Representative FACS profiles and bar graphs are shown and numbers correspond to percentages of cells positively stained with the H-2K^b^/Ova_257–264_ pentamer in the gated CD8^+^ population. (**B**) 2×10^7^ purified CD8^+^ T cells originating from B6 or SM-Ova donor mice were adoptively transferred into syngenic CD3^−/−^ recipient mice (n = 6) together with 3×10^7^ purified CD4^+^ T cells originating exclusively from B6 donors. Recipient CD3^−/−^ mice were immunized with rAAV-Ova one day later and splenocytes were collected, enumerated and analyzed 14 days after by flow cytometry to evaluate the percentages and absolute numbers of Ova-specific CD8^+^ cells. Representative profiles gated on the CD8^+^ population are shown in the left panel and the total numbers of Ova-specific CD8^+^ per spleen are shown in the right bar graph.

As regulatory T cells (Treg) naturally exert suppressive functions on priming and proliferation of autoreactive T cells, we investigated whether they could be responsible for the inhibition of Ova-specific CD8^+^ T cell responses observed in SM-Ova mice. For that, mice were treated with the anti-CD25 antibody (PC-61) using a protocol widely used to deplete Treg *in vivo*. We have previously shown that this protocol is sufficient to promote a spontaneous anti-tumor immune response against Ova-expressing EG-7 cells [19]. Here, Treg depletion before immunization did not restore the capacity of SM-Ova mice to mount an Ova-specific CD8^+^ T cell response (Fig. 2A).

However, anti-CD25 antibody treatment may also deplete activated conventional T cells as for instance endogenous CD8^+^ T cells activated in vivo by Ova-antigen encounter. Therefore, to further exclude the involvement of Ova-specific Treg, we adoptively transferred purified naïve CD8^+^ lymphocytes from SM-Ova mice into lymphopenic CD3^−/−^ syngenic recipients before an Ova challenge. In these experiments, sorted CD4^+^ lymphocytes from naïve B6 mice were co-transferred together with purified CD8^+^ lymphocytes to provide optimal CD4 help. The results showed that, even in the T cell deficient environment of CD3^−/−^ mice, the CD8^+^ T cells purified from SM-Ova mice - and consequently devoid of CD4^+^ Treg from this donor - remained unable to respond to Ova-immunization (Fig. 2B). Hence, dominant suppressive mechanisms by CD4^+^ T cells from SM-Ova mice are not primarily involved in the tolerance mechanisms at study. Instead, this suggested that Ova-specific CD8^+^ precursors from SM-Ova had been deleted from the peripheral lymphocytic pool of SM-Ova animals.

### Deletion of adoptively transferred OT-I cells in SM-Ova mice

**Figure 3:**
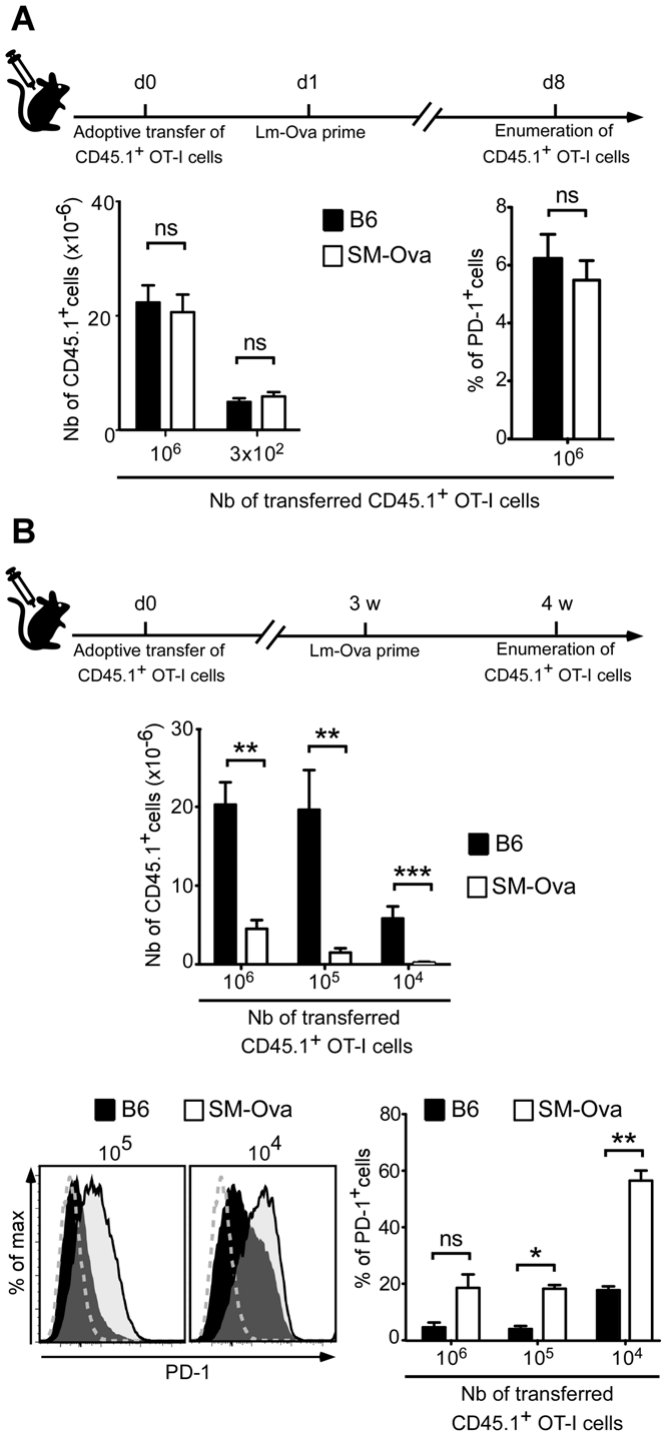
Adoptively transferred OT-I CD8^+^ T cells are tolerized in SM-Ova recipient mice. Indicated numbers of purified CD8^+^ T cells from CD45.1^+^ OT-I mice were adoptively transferred into B6 or SM-Ova mice. Recipients were immunized one day later (**A**) or 3 weeks later (**B**) with Lm-Ova and splenocytes were collected, enumerated and analyzed 7 days after immunization by flow cytometry. Bar graphs show the total numbers of CD8^+^CD45.1^+^ cells per spleen and the mean percentage of cells expressing PD-1 in the CD8^+^CD45.1^+^ gated population. Representative flow cytometry overlay profiles are shown in (**B**), illustrating the intensity of PD-1 staining in the CD8^+^CD45.1^+^ gated cells recovered from B6 (black histograms) or SM-Ova mice (open histograms) when 10^5^ or 10^4^ OT-I cells were adoptively transferred. Negative staining controls obtained with an isotype-matched irrelevant antibody are also shown (dashed grey lines). Data are representative of 2 independent experiments each one performed with 5–8 mice per group.

To evaluate more directly how the CD8 compartment is tolerized in SM-Ova mice, we adoptively transferred purified Ova-specific CD8^+^ T cells from TCR-Tg OT-I mice harboring the CD45.1^+^ allelic variant, and monitored their response to an Ova challenge *in vivo* in the environment of SM-Ova mice. Transfer of CD8^+^ OT-I T cells followed 1 day after by immunization with Lm-Ova led to a comparable level of CD8^+^ OT-I cell expansion in B6 and SM-Ova recipient mice (Fig. 3A). This further confirmed that Ova-specific CD8^+^ immune responses are not persistently inhibited in SM-Ova mice by a dominant mechanism of suppression.

We next evaluated if naïve CD8^+^ from OT-I mice were gradually deleted after their transfer to SM-Ova recipients. For that, purified CD8^+^ OT-I T cells were again adoptively transferred in either B6 or SM-Ova recipients but immunization with Lm-Ova vaccine was performed only after a lag period of 3 weeks. In this experimental setting, much fewer CD8^+^ OT-I T cells could be detected in the spleens of SM-Ova mice after immunization as compared to B6 controls (Fig. 3B). Of note, the few remaining CD8^+^ OT-I T cells detected in the SM-Ova recipient had up-regulated the immunoregulatory PD-1 receptor on their surface (Fig. 3B). These data suggested that Ova-reactive CD8^+^ T cells are progressively deleted in the context of the SM-Ova environment.

**Figure 4:**
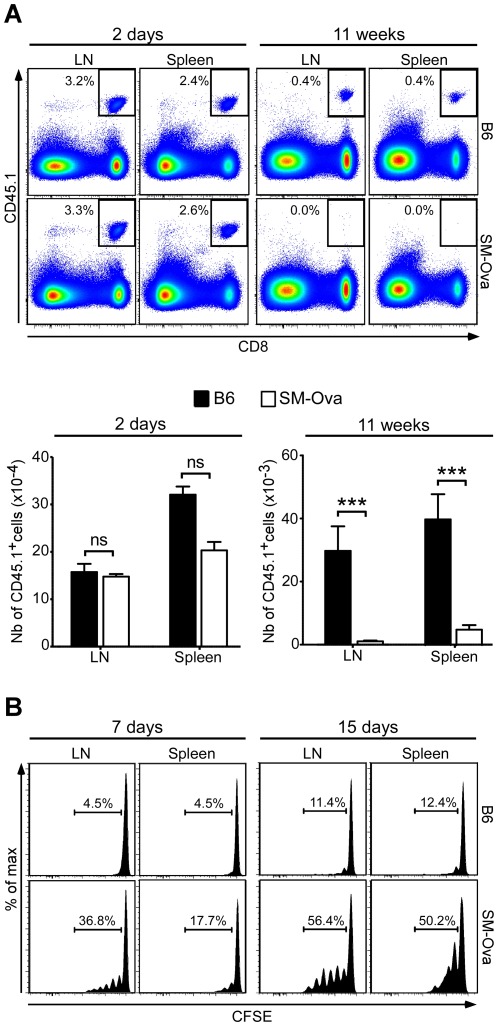
Adoptively transferred OT-I CD8^+^ T cells are deleted in SM-Ova mice. (**A**) 5×10^6^ purified CD8^+^CD45.1^+^ OT-I cells were adoptively transferred into SM-Ova or control B6 mice. Recipients were euthanized 2 days or 11 weeks after to determine the percentages (indicated in the representative flow cytometry panels on top) and total numbers (represented below in the bar graphs) of CD8^+^CD45.1^+^ OT-I cells recovered from the LNs and spleen of recipient mice. (**B**) 5×10^6^ CFSE-labeled CD8^+^CD45.1^+^ OT-I cells were injected i.v. into SM-Ova or B6 control mice. Proliferation of gated CD8^+^CD45.1^+^ cells was evaluated in the LN and spleen of recipient mice by flow cytometry 7 or 15 days after their adoptive transfer. Representative profiles are shown and the indicated numbers correspond to the percentage of gated cells that have diluted CFSE upon cell division. Data are representative of 2 independent experiments performed with 10 mice per group.

To formally establish the involvement of peripheral deletion in tolerance of CD8^+^ T cells, we adoptively transferred a high number of CD8^+^ OT-I cells (i.e., 5×10^6^ cells) and directly monitored their survival over time in the absence of Ova immunization. For that we counted the numbers of remaining CD8^+^ cells that express the CD45.1 allelic marker in spleens and LNs. As a result we observed a significant loss of CD8^+^ OT-I T cells over time in unimmunized SM-Ova mice, with their almost complete disappearance 11 weeks after transfer (Fig. 4A). This markedly contrasted with the results observed in B6 recipients in which higher numbers of Ova-specific CD8^+^ T cells persisted overtime. These data unequivocally establish that Ova-specific CD8^+^ T cells are peripherally deleted from the repertoire of SM-Ova mice.

### Autoreactive CD8^+^ T cells are exposed to Ova antigens in the lymph nodes of SM-Ova mice

Several studies previously suggested that peripheral deletion of self reactive CD8^+^ T cells is mediated by dendritic cells migrating from tissues to regional LNs where cell-derived self antigens are cross-presented [20], [21]. Therefore, we tested whether Ova-reactive T cells from a non tolerant OT-I donor could respond to endogenous Ova when transferred to SM-Ova mice. For that, CFSE-labeled CD8^+^ OT-I cells were transferred to SM-Ova mice in order to evaluate their proliferation in lymphoid organs. Not surprisingly, a fraction of the OT-I T cells underwent 1 to 6 cycles of division over a period of 7–15 days post-transfer in SM-Ova mice while OT-I cells transferred in B6 controls remained mostly undivided (Fig. 4B). Importantly also, transferred cells appeared to proliferate earlier and more vigorously in the lymph nodes (i.e., which drain muscle) as compared to the spleen (i.e., which does not drain muscle). These results indicate that OT-I CD8^+^ T cells respond *in vivo* to their muscle-derived cognate autoantigen in LNs of SM-Ova mice.

### Deletion of peripheral CD8^+^ T cells in thymectomized [SM-Ova×OT-I]F1 mice

**Figure 5:**
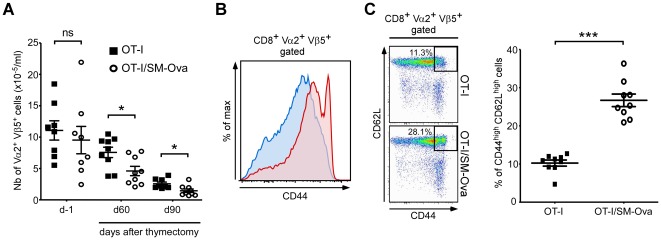
Peripheral deletion of Ova-specific CD8^+^ can be evidenced in double transgenic [OT-I×SM-Ova]F1 mice after thymectomy. (**A**) Double transgenic [OT-I×SM-Ova]F1 and control OT-I mice were thymectomized at day 0. The total numbers of Vα2^+^Vβ5^+^CD8^+^ cells were determined from blood samples one day before thymectomy and 60 or 90 days after thymectomy. (**B**) Representative flow cytometry overlay profile illustrating the level of CD44 expression in peripheral blood cells from unmanipulated [OT-I×SM-Ova]F1 or OT-I control mice in the gated Vα2^+^Vβ5^+^CD8^+^ population. (**C**) Representative flow cytometry profiles and a scatter plot graph illustrating the percentage of CD44^high^CD62L^high^ cells found in blood samples from unmanipulated [OT-I×SM-Ova]F1 or OT-I control mice in the gated Vα2^+^Vβ5^+^CD8^+^ population. Data are representative of 2 independent experiments with 8–9 mice per group.

We have previously noticed that the number of peripheral Vα2^+^CD8^+^ T cells in [SM-Ova×OT-I]F1 double Tg mice was not significantly different from that found in OT-I control mice [15]. This was previously interpreted as a lack of peripheral deletion. In light of our present findings, we revisited the possibility of peripheral deletion in this double Tg model. We reasoned that cell deletion is presumably compensated in this double Tg model by the continuous output of high numbers of Ova-specific CD8^+^ T cells from the thymus. This hypothesis would imply that the number of Vα2^+^CD8^+^ cells in [SM-Ova×OT-I]F1 double Tg mice should decrease after thymectomy. Indeed, results showed a significantly faster decrease in the absolute number of Vα2^+^Vβ5^+^CD8^+^ T cells over time in the periphery of thymectomized double Tg mice as compared to thymectomized OT-I controls (Fig. 5A). Interestingly, a higher proportion of Vα2^+^Vβ5^+^CD8^+^CD44^high^CD62L^high^ cells were found in double Tg mice before thymectomy (Fig. 5B). This indicated that a significant fraction of Ova-specific CD8^+^ T cells had been partially activated by encountering Ova antigen *in vivo* and are presumably engaged into a deletional pathway. However, the fate of these subsets could not be followed after thymectomy since, in both groups of mice, the majority of CD8^+^ cells progressively acquired a CD44^high^ phenotype most probably as the result of homeostatic expansion that compensates for the arrest of thymic production (data not shown).

**Figure 6:**
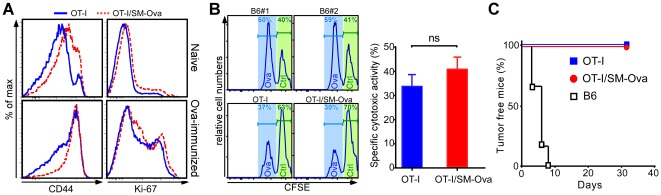
CD8^+^ cells from [SM-Ova×OT-I]F1 double Tg mice are not functionally defective. (**A**) Double Tg [SM-Ova×OT-I]F1 mice (n = 3) and control OT-I mice (n = 3) were either left unmanipulated or immunized i.v. with an Ova-encoding lentiviral vector as described [37]. Representative flow cytometry overlay profiles illustrate the level of CD44 or Ki-67 in the gated CD8^+^Vα2^+^ T cells population collected from the spleens of OT-I (blue histograms) or [SM-Ova×OT-I]F1 (red dashed histograms) mice 7 days later. (**B**) Naive double transgenic [SM-Ova×OT-I]F1 (n = 8), OT-I mice (n = 8) and naive control B6 mice (n = 2) were injected i.v with a 1∶1 mixture of CFSE^high^ unpulsed cells and CFSE^low^ SIINFEKL pulsed cells as described [15]. Spleens were harvested 5 hours later and analyzed for detection of CFSE-labeled cells. *In vivo* specific cytotoxic activities were determined by calculating the percentage of specific lysis, as described [15]. (**C**) Naive [SM-Ova×OT-I]F1 (n = 5), control OT-I mice (n = 5) mice and naive B6 mice (n = 6) were inoculated with 3×10^6^ Ova-bearing EG-7 tumor cells and monitored during 30–40 days for tumor development, as described [19].

Noteworthy, in this TCR-Tg model where large numbers of OT-I cell are present in the periphery, CD8^+^ responses to an Ova challenge were normal as illustrated by i) the up-regulation of CD44 on the majority of CD8^+^Vα2^+^ lymphocytes (Fig. 6A), ii) the prominent *in vivo* cytotoxic activity against SIINFEKL-pulsed target cells (Fig. 6B) and iii) the rejection of Ova-bearing tumor cells even in naïve [SM-Ova×OT-I]F1 mice (Fig. 6C). All together these results demonstrate that the CD8^+^ compartment respond normally to an Ova challenge in the double Tg [SM-Ova×OT-I]F1 mice and that the peripheral deletion is not able to tolerize the CD8^+^ compartment in this repertoire-biased model where virtually all CD8^+^ cells are directed against Ova.

### Peripheral deletion of endogenously produced Ova-specific CD8^+^ T cells in SM-Ova

**Figure 7:**
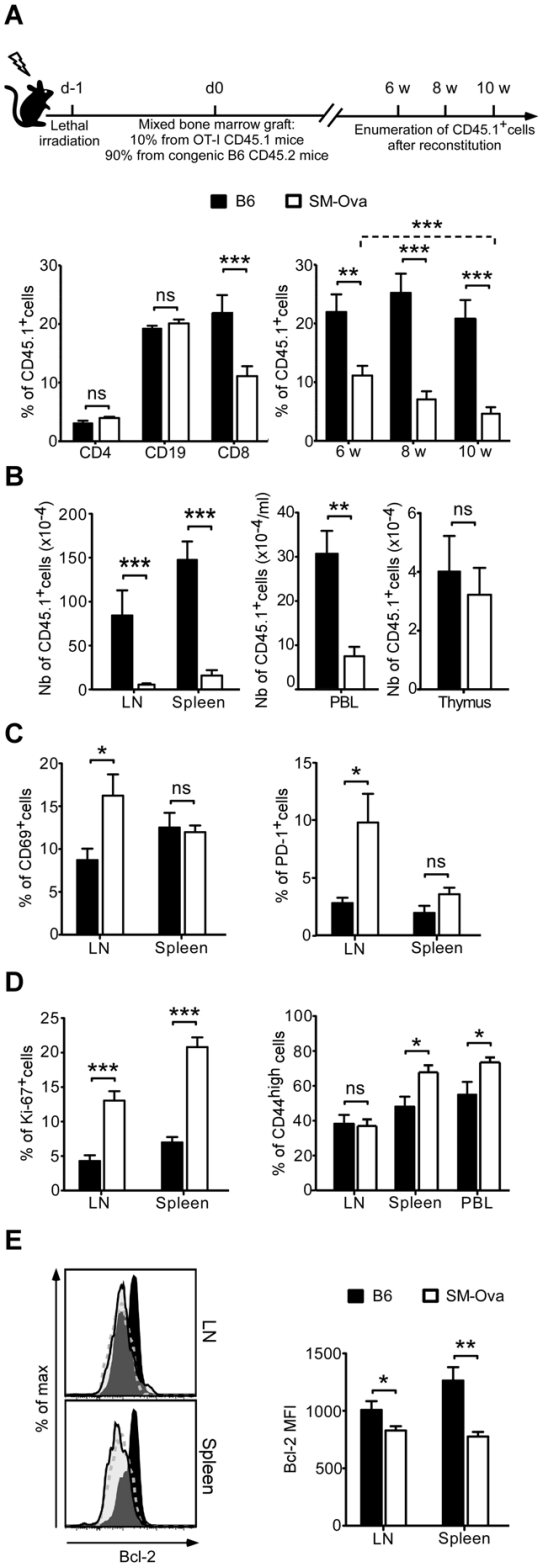
Peripheral deletion of endogenously produced Ova-specific CD8^+^ T cells in chimeric SM-Ova mice. B6 (n = 8) and SM-Ova (n = 13) recipient mice were lethally irradiated and reconstituted one day after with bone marrow cells collected from B6 donor mice mixed with 10% bone marrow cells collected from CD45.1^+^ OT-I donor mice. (**A**) Left bar graph illustrates the percentages of cells in the CD4^+^, CD19^+^ or CD8^+^ gated populations expressing the CD45.1 marker in blood samples from B6 (black bars) or SM-Ova (open bars) recipient mice 6 weeks after bone marrow transplantation. Right bar graph illustrates the percentage of CD8^+^ PBL expressing the CD45.1 marker 6, 8 or 10 weeks after bone marrow transplantation. (**B**) Absolute numbers of CD8^+^ CD45.1^+^ OT-I T cells found in LN, spleen, blood and thymus of chimeric mice were enumerated and analyzed by flow cytometry 10 weeks after bone marrow engraftment. (**C, D**) Bar graphs show the percentages of cells expressing CD69, PD-1, CD44 or Ki-67 marker in the gated CD8^+^CD45.1^+^ population. (**E**) Representative flow cytometry overlay profiles illustrate the level of Bcl-2 expression in the gated CD8^+^CD45.1^+^ T cells population collected from the LN and spleen of B6 (dark histograms) or SM-Ova (open histograms) recipient mice 10 weeks after bone marrow transplantation. Isotype-matched negative staining controls are also shown (dashed grey lines). The corresponding bar graph illustrating the mean fluorescence intensities (MFI) of Bcl-2 expression is shown in the right panel.

Our results so far have demonstrated that adoptively-transferred exogenous Ova-reactive T cells are progressively deleted in the periphery of SM-Ova mice and therefore indirectly suggest that endogenously produced Ova-specific CD8^+^ are also tolerized by a mechanism of peripheral deletion. To definitively establish this point, we turned to bone marrow chimera experiments as they represent the best model to directly study the fate of endogenously-produced Ova-specific CD8^+^ T cells in the context of a polyclonal repertoire and also offer the possibility to examine their differentiation in the thymus. For this, lethally-irradiated SM-Ova mice were reconstituted with a 9∶1 mixture of bone marrow cells from B6 and CD45.1^+^ OT-I mice, respectively. After reconstitution in SM-Ova mice, the fate of emerging CD45.1^+^ cells was monitored over time from blood samples, and then analyzed in central and peripheral organs after sacrificing the mice. Results demonstrated that the frequency of CD8^+^CD45.1^+^ cells detected in the circulation of SM-Ova mice between 6 and 10 weeks after reconstitution gradually decreased as compared to B6 recipients (Fig. 7A, right panel). In contrast, CD4^+^CD45.1^+^ T lymphocytes and CD19^+^CD45.1^+^ B lymphocytes remained in similar frequencies in B6 and SM-Ova mice (Fig. 7A, left panel). Ten weeks after reconstitution, the absolute numbers of Ova-specific CD8^+^CD45.1^+^ T cells were reduced by 75 to 94% in the LNs, spleen and blood of SM-Ova chimeric mice, demonstrating their peripheral deletion (Fig. 7B). The situation was different in the thymus where CD8^+^CD45.1^+^ T cells were found in similar numbers in SM-Ova as compared to B6 recipient mice (Fig. 7B, right panel), indicating that deletion of Ova-specific CD8^+^ T cells was only peripheral but not central.

Phenotypic analysis of the remaining peripheral CD8^+^CD45.1^+^ cells further evidenced a marked increase in the percentage of cells expressing the early activation marker CD69 and the immunoregulatory protein PD-1 in the LNs of SM-Ova mice as compared to B6 mice (Fig. 7C). This was not the case for cells harvested from the spleens of SM-Ova mice, indicating that Ova-specific CD8^+^ T cells encounter their cognate antigen in the muscle-draining LNs rather than in the spleen. CD8^+^CD45.1^+^ cells expressing CD44^high^, a late marker of cell activation, were instead found at higher frequencies in the spleens and in the circulation of SM-Ova mice (Fig. 7D, right panel), suggesting that these T cells rapidly recirculate after having encountered their auto-antigen and preferentially relocalize to the spleen. Apart from the phenotypic changes, CD8^+^CD45.1^+^ T cells also proliferate more intensely in SM-Ova mice, as evidenced by the higher frequency of cells expressing the nuclear protein Ki-67 characteristic of cell division (Fig. 7D, left panel). Finally, CD8^+^CD45.1^+^ T cells remaining in SM-Ova mice were found to express significantly lower levels of the anti-apoptotic protein Bcl-2, suggesting that their probability of survival was indeed hindered in the presence of their muscle-expressed cognate autoantigen *in vivo* (Fig. 7E).

## Discussion

Muscle represents a major constituent of the body, yet the precise mechanisms governing immune priming/tolerance toward muscle antigens are still poorly defined. In this study, we examined how immunological tolerance is established in the CD8^+^ T cell compartment of SM-Ova Tg mice that express an Ova neo-autoantigen specifically in skeletal muscle. We previously demonstrated that CD4^+^ T cell as well as B cell responses can be readily induced in SM-Ova single Tg mice after immunization with Ova, indicating that immunological ignorance of muscle-derived autoantigens rather than tolerance prevails in the steady state for these lymphocyte subsets [15]. In contrast, Ova-specific CD8^+^ T cells responses were not detected suggesting that a different mechanism of tolerance controls the emergence of muscle autoreactive CTLs. Since the number of CD8^+^ T cells was found to be normal in the double Tg [OT-I×SM-Ova]F1 mice, Ova-specific CD8^+^ T cells defect in SM-Ova models was interpreted as being possibly associated with a functional defect. We demonstrate herein that muscle-reactive CD8^+^ T cells are in fact deleted from the peripheral T cell repertoire of single transgenic SM-Ova mice, a phenomenon that is not apparent in the double Tg [OT-I×SM-Ova]F1 mice.

Several observations in this report demonstrate that peripheral deletion is the central mechanism of CD8^+^ T cell tolerance in SM-Ova mice: i) the absence of detectable CD8^+^ lymphocytes specific for H-2K^b^/Ova_257–264_, even upon challenge with strongly immunogenic rAAV-Ova, Lm-Ova or VSV-Ova vaccines (Fig. 1), ii) the gradual loss of exogenous CD8^+^ T cells from OT-I mice adoptively transferred to SM-Ova (Fig. 3 and 4), and iii) the peripheral loss overtime of endogenously-produced Ova-specific CD8^+^ T cells in chimeric SM-Ova mice engrafted with bone marrow cells partly derived from OT-I mice (Fig. 7). Noteworthy, in the latter SM-Ova chimeric mice, the loss of Ova-specific CD8^+^ T cells could only be detected in the peripheral lymphoid compartments but not in the thymus, confirming the absence of central deletion (Fig. 7B, right panel) which is consistent with the absence of Ova-transgene expression in the thymus of SM-Ova mice [15].

Peripheral deletion of autoreactive CD8^+^ T cells was not evidenced in double Tg [OT-I×SM-Ova]F1 mice as compared to single Tg OT-I control mice (Fig. 5A, day −1). This observation confirms that peripheral deletion in this context is masked at least partially by the continuous production of thymic-derived TCR-Tg T cells. This view is further supported by the observation that autoreactive CD8^+^Vα2^+^Vβ5^+^ lymphocytes decline more rapidly overtime in thymectomized [OT-I×SM-Ova]F1 mice than in thymectomized OT-I control mice (Fig. 5A). This underscores that TCR/antigen double Tg mice do not represent appropriate models to study peripheral deletion. Instead, as used here, chimeric mice reconstituted with a mixture of bone marrow cells composed by 90% of cells originating from control mice and by only 10% of cells from the TCR-Tg mice (i.e., OT-I mice) allowed to more closely model the situation where endogenously produced lymphocytes specific for a given antigen represent only a minority of the T cell repertoire. In these chimeric mice, peripheral deletion of Ova-specific CD8^+^ cells is readily demonstrable (Fig. 7), probably because deleted cells are progressively replaced by polyclonal non-Tg TCR lymphocytes that may display a better fitness in situation of interclonal competition [22], [23].

From the present data, it is possible to approximate the rate at which peripheral deletion affects autoreactive CD8^+^ T cells. Around 1×10^3^ Ova-specific CD8^+^ cells could be detected in LNs of SM-Ova mice 11 weeks post-transfer, as compared to approximately 3×10^4^ cells in control mice (Fig. 4A, left panel). Therefore, a little bit less than 3×10^3^ cells are deleted per week. This is consistent with the fact that transfer of less than 1×10^4^ cells resulted in a complete deletion of transferred cells 3 weeks later, as attested by our inability to detect their expansion upon Lm-Ova challenge (data not shown, similar to experiments presented in Fig. 3). This may further explain our inability to evidence the peripheral deletion in [OT-I×SM-Ova]F1 double transgenic mice as thymic output has been estimated to range between 10^4^–10^6^ cells per day depending on age [24]. Noteworthy, this rate of peripheral deletion is still largely sufficient for the complete attrition of all Ova-specific CD8^+^ cells in single Tg SM-Ova mice, since the total number of CD8^+^ cells directed against a given antigenic epitope in naive mice has been estimated to be around 200 cells [25].

As we used H-2K^b^/Ova_257–264_ pentamer staining to detect Ova-specific CD8^+^ cells directed against the immunodominant SIINFEKL peptide and the high affinity OT-I cells in our adoptive transfer experiments, the question may arise as whether lower affinity Ova-specific CD8^+^ T cells are possibly preserved or also peripherally deleted in SM-Ova mice. The fact that SM-Ova mice immunized with strongly immunogenic vaccines encoding the entire Ova protein were unable to reject an Ova-bearing tumor (Fig. 1A) suggests that lower affinity Ova-specific CD8^+^ T cells are either also absent or unable to mediate an efficient cytotoxic immune response against Ova-expressing cells.

Another important question concerns the location where Ova-derived antigens are presented to autoreactive CD8^+^ T cells. From the results of CFSE-labeled OT-I T cell transfer experiments, it is likely that antigen encounter occurs in the LNs rather than in the spleen (Fig. 4B). In agreement, we also found in the chimeric model that OT-I derived CD8^+^ T cells upregulated the early activation markers CD69 and PD-1 in the LNs but not in the spleen (Fig. 7C).Yet, we observed a higher fraction of cells displaying the late activation marker CD44^high^ in the spleen as compared to the LNs (Fig. 7D). Since CD44^high^ lymphocytes home more efficiently to the spleen than to the LNs [26], these data are compatible with a sequence of events where Ova-specific CD8^+^ T cells first encounter their cognate autoantigen in the LNs draining muscle territories, rapidly upregulate CD69 and PD-1, proliferate, acquire the CD44^high^ marker, relocalize preferentially to the spleen and finally die after having down-regulated the anti-apoptotic factor Bcl-2 (Fig. 4 and 7). Induction of cell death in this situation most probably involves partial T cell activation in absence of costimulatory molecules at the surface of APCs and paucity of cytokine secretion in non inflammatory conditions [21], [27]–[29]. Recent work has shown that not only lack of accessory molecules but also presence of inhibitory signals can promote tolerization [30]. One such signal could be delivered to PD-1-expressing lymphocytes by PD-L1 or PD-L2 ligands expressed by APCs. Indeed, numerous studies have now recognized the PD-1 pathway as an essential molecular trigger involved in the maintenance of tolerance to tissue antigens [30], [31]. Here, PD-1 is expressed by a significant fraction of Ova-specific CD8^+^ T cells of SM-Ova mice (Fig. 3B, 4C and 7C). Therefore, it is conceivable that PD-1 participates in induction of tolerance and possibly in induction of cell death. Such a view would be in agreement with the finding that PD-1 upregulation has been recently found to represent one of the key molecular signature of CD8^+^ T cells undergoing deletional tolerance, together with the downregulation of Bcl-2 and IL-7α expression [32]. In line with this, we recently reported that that PD-L1 gene transfer protects muscle from CD8^+^ cytotoxicity in gene therapy settings [16].

A puzzling feature in the SM-Ova model is the inability to induce myositis upon transfer of OT-I cells and immunization, even if the transferred cells can reject Ova-bearing tumors (data not shown). To our knowledge, in all other transgenic models where Ova is expressed in tissues such as pancreas, intestine, skin or prostate, the adoptive transfer of OT-I T cells, either activated *in vitro* or *in vivo* by immunization with VSV-Ova or Lm-Ova live vectors, has led to cell infiltration and autoimmunty directed against the Ova-expressing tissue [17], [33]–[35]. Therefore, other muscle-specific mechanisms may be at play to control T cell-mediated cytotoxicity against this tissue. Noteworthy, muscle fibers do not physiologically express MHC-I molecules and are therefore intrinsically resistant to CD8^+^ T cells autoimmune aggression [2]–[5]. In human polymyositis, MHC-I is upregulated at the surface of myofibers and the presence of MHC-I-expressing myofibers surrounded by CD8^+^ lymphocytes is one of the hallmarks and diagnosis criteria of polymyositis [10]–[12]. Future experimental studies should address whether induction of stable MHC-I expression in muscle by gene transfer could lead to the breakage of tolerance in this model.

In summary, we show herein that tolerance toward an autoantigen expressed in skeletal muscles involves the deletion of high affinity autoreactive CD8^+^ T cells from the peripheral repertoire, a mechanism that prevents the generation of CTLs upon immune challenge with the same antigen. These results shed light on the mechanisms governing immune tolerance toward muscle antigens and could be of importance to better understand the pathogenesis of muscle autoimmune diseases.

## Materials and Methods

### Mice

Transgenic SM-Ova mice, expressing a membrane-bound form of ovalubumin (Ova) exclusively in skeletal muscle have been described previously [15]. In some experiments, SM-Ova mice were crossed for one generation with TCR-Tg OT-I mice harboring large numbers of CD8^+^ T cells that specifically recognize the Ova_257–264_ peptide in the context of MHC-I (H-2K^b^) presentation. OT-I, C57BL/6 (B6) and congenic B6 mice harboring the CD45.1 allelic marker were obtained from Charles River Laboratories. CD3ε^−/−^ mice were obtained from “Centre de Développement des Techniques Avancées" (CDTA, Orléans, France). Mice were housed in a specific pathogen-free barrier facility and were analyzed between 8–16 weeks of age. Animal experiments were approved by the local institutional ethic committee for animal experimentation (authorization #0211-22 “Comité Régional d'Éthique en Expérimentation Animale de Normandie").

### Reagents, antibodies, flow cytometry and Treg depletion

Fluorochrome-conjugated antibodies to mouse CD4, CD8, CD45.1, CD44, CD62L, PD-1, CD69 and unlabeled CD16/CD32 antibodies were obtained from eBioscience. Fluorescently labeled anti- Vα2, Vβ5, Ki-67 and Bcl-2 were purchased from BD Biosciences. Intracellular Ki-67 and Bcl-2 staining were performed according to the manufacturer's protocol. PE-conjugated H-2K^b^/Ova_257–264_ or H2-K^b^/VSV pentamers were used to detect CD8^+^ cells that specifically recognize the immunodominant SIINFEKL peptide from Ova or the RGYVYQGL peptide from VSV-nucleoprotein using the manufacturer's protocol (ProImmune). Flow cytometry measurements of single-cell suspensions derived from spleen, peripheral blood (PBL), thymus or pooled lymph nodes (LNs) (i.e., maxillary, axillary, brachial, inguinal, para-aortic and popliteal lymph nodes) were performed using standard procedures on a FACSCanto (BD Biosciences) and analyses were made using the FlowJo software (Tree Star). In some experiments, the monoclonal antibody PC-61 directed against CD25 was used to inactivate CD4^+^CD25^+^ Treg *in vivo*. For that, 250 µg of purified antibody (BioXcell) were injected i.p. at day −4 and at day −1 as described previously [19].

### Mice immunization

Replicative vesicular stomatitis virus (VSV) encoding Ova (VSV-Ova) [17] and a live strain of *Listeria monocytogenes* expressing Ova (Lm-Ova) [18] were kindly provided by L. Lefrançois and G. Lauvau, respectively. Where indicated, B6 or SM-Ova mice were injected i.v. with 1×10^6^ pfu VSV-Ova or Lm-Ova preparations diluted in 100 µl of PBS. Immune responses were evaluated 7 days after infection. The plasmid construct for the preparation of recombinant adeno-associated virus encoding for soluble Ova (rAAV-Ova) [36] was kindly provided by R. Herzog. Where indicated, anesthetized B6 or SM-Ova mice were injected i.m. with 1×10^11^ equivalent vector genomes of rAAV2/1-Ova preparations diluted in 50 µl of PBS and analyzed 14 days after.

### Adoptive T cell transfer

Ova-specific CD8^+^ T cells were obtained from the LNs of CD45.1^+^ TCR-Tg OT-I mice and purified by magnetic sorting using a CD8^+^ negative isolation kit (Invitrogen). Between 92 and 96% of the purified cells displayed a CD8^+^Vα2^+^CD45.1^+^CD4^−^CD19^−^ phenotype as assessed by flow cytometry. Indicated numbers of purified OT-I T cells were then adoptively transferred into SM-Ova or B6 recipients by i.v. injection and, where indicated, recipient mice were infected 1 day or 3 weeks later by Lm-Ova. For *in vivo* proliferation assays, purified 5×10^6^ CD45.1^+^ OT-I T cells were stained with 5 µM CFSE (Invitrogen) for 10 min at 37°C, washed and injected i.v. into SM-Ova or B6 recipient mice. For adoptive T cell transfer into CD3^−/−^ recipient mice, 2×10^7^ CD8^+^ T cells derived from the LNs and spleen of either SM-Ova or B6 donor mice were purified using magnetic sorting and then injected i.v. into CD3^−/−^ recipient mice together with 3×10^7^ magnetically purified CD4^+^ helper T cells derived exclusively from B6 donors.

### Tumor model

B6 or SM-Ova mice, immunized 7 days before with VSV-Ova or Lm-Ova, or 14 days before with rAAV-Ova, were injected s.c. in their shaved flanks with 1×10^6^ EL4-Ova (EG-7) tumor cells obtained from subconfluent *in vitro* cultures. Mice were then examined every third day and tumor development was monitored for 30–40 days as described [19].

### Bone-marrow chimeras

Bone marrow transplantation was performed on B6 or SM-Ova mice at 8 weeks of age. For that, B6 or SM-Ova mice were lethally irradiated on day 0 by administration of 2 cumulative doses of 5 Gy each separated by a 3 hours rest period using a Faxitron CP225 X-ray cabinet set at 220 kV and 10 mA. Irradiated recipients were reconstituted one day after by i.v. injection of 9×10^6^ bone marrow cells collected from B6 mice mixed with 1×10^6^ bone marrow cells from CD45.1^+^ OT-I mice.

### Data representation and statistical analysis

All data are shown as mean values and error bars represent SEM. Parametric tests were used for statistical comparison between experimental groups using one-way analysis of variance (ANOVA). Differences were considered statistically significant when p values were less than 0.05(*), 0.01 (**) or 0.001 (***). All calculations were performed using the Prism software (GraphPad).

## References

[1] Goebels N, Michaelis D, Wekerle H, Hohlfeld R (1992). Human myoblasts as antigen-presenting cells.. J Immunol.

[2] Emslie-Smith AM, Arahata K, Engel AG (1989). Major histocompatibility complex class I antigen expression, immunolocalization of interferon subtypes, and T cell-mediated cytotoxicity in myopathies.. Hum Pathol.

[3] McDouall RM, Dunn MJ, Dubowitz V (1989). Expression of class I and class II MHC antigens in neuromuscular diseases.. J Neurol Sci.

[4] van der Pas J, Hengstman GJ, ter Laak HJ, Borm GF, van Engelen BG (2004). Diagnostic value of MHC class I staining in idiopathic inflammatory myopathies.. J Neurol Neurosurg Psychiatry.

[5] Englund P, Lindroos E, Nennesmo I, Klareskog L, Lundberg IE (2001). Skeletal muscle fibers express major histocompatibility complex class II antigens independently of inflammatory infiltrates in inflammatory myopathies.. Am J Pathol.

[6] Murata K, Dalakas MC (1999). Expression of the costimulatory molecule BB-1, the ligands CTLA-4 and CD28, and their mRNA in inflammatory myopathies.. Am J Pathol.

[7] Wiendl H, Mitsdoerffer M, Schneider D, Chen L, Lochmuller H (2003). Human muscle cells express a B7-related molecule, B7-H1, with strong negative immune regulatory potential: a novel mechanism of counterbalancing the immune attack in idiopathic inflammatory myopathies.. FASEB J.

[8] Marino M, Scuderi F, Provenzano C, Bartoccioni E (2011). Skeletal muscle cells: from local inflammatory response to active immunity.. Gene Ther.

[9] Arnold L, Henry A, Poron F, Baba-Amer Y, van Rooijen N (2007). Inflammatory monocytes recruited after skeletal muscle injury switch into antiinflammatory macrophages to support myogenesis.. J Exp Med.

[10] Cox S, Limaye V, Hill C, Blumbergs P, Roberts-Thomson P (2010). Idiopathic inflammatory myopathies: diagnostic criteria, classification and epidemiological features.. Int J Rheum Dis.

[11] Dalakas MC (2010). Inflammatory muscle diseases: a critical review on pathogenesis and therapies.. Curr Opin Pharmacol.

[12] Dalakas MC (2010). Immunotherapy of myositis: issues, concerns and future prospects.. Nat Rev Rheumatol.

[13] Bender A, Ernst N, Iglesias A, Dornmair K, Wekerle H (1995). T cell receptor repertoire in polymyositis: clonal expansion of autoaggressive CD8+ T cells.. J Exp Med.

[14] Benveniste O, Cherin P, Maisonobe T, Merat R, Chosidow O (2001). Severe perturbations of the blood T cell repertoire in polymyositis, but not dermatomyositis patients.. J Immunol.

[15] Calbo S, Delagreverie H, Arnoult C, Authier FJ, Tron F (2008). Functional tolerance of CD8+ T cells induced by muscle-specific antigen expression.. J Immunol.

[16] Adriouch S, Franck E, Drouot L, Bonneau C, Jolinon N (2011). Improved immunological tolerance following combination therapy with CTLA-4/Ig and AAV-mediated PD-L1/2 muscle gene transfer.. Front Microbiol.

[17] Vezys V, Olson S, Lefrancois L (2000). Expression of intestine-specific antigen reveals novel pathways of CD8 T cell tolerance induction.. Immunity.

[18] Lauvau G, Vijh S, Kong P, Horng T, Kerksiek K (2001). Priming of memory but not effector CD8 T cells by a killed bacterial vaccine.. Science.

[19] Hubert S, Rissiek B, Klages K, Huehn J, Sparwasser T (2010). Extracellular NAD+ shapes the Foxp3+ regulatory T cell compartment through the ART2-P2X7 pathway.. J Exp Med.

[20] Kurts C, Cannarile M, Klebba I, Brocker T (2001). Dendritic cells are sufficient to cross-present self-antigens to CD8 T cells in vivo.. J Immunol.

[21] Lutz MB, Kurts C (2009). Induction of peripheral CD4+ T-cell tolerance and CD8+ T-cell cross-tolerance by dendritic cells.. Eur J Immunol.

[22] Freitas AA, Agenes F, Coutinho GC (1996). Cellular competition modulates survival and selection of CD8+ T cells.. Eur J Immunol.

[23] Leitao C, Freitas AA, Garcia S (2009). The role of TCR specificity and clonal competition during reconstruction of the peripheral T cell pool.. J Immunol.

[24] Berzins SP, Boyd RL, Miller JF (1998). The role of the thymus and recent thymic migrants in the maintenance of the adult peripheral lymphocyte pool.. J Exp Med.

[25] Blattman JN, Antia R, Sourdive DJ, Wang X, Kaech SM (2002). Estimating the precursor frequency of naive antigen-specific CD8 T cells.. J Exp Med.

[26] Williams MB, Butcher EC (1997). Homing of naive and memory T lymphocyte subsets to Peyer's patches, lymph nodes, and spleen.. J Immunol.

[27] Kurts C, Robinson BW, Knolle PA (2010). Cross-priming in health and disease.. Nat Rev Immunol.

[28] Miller JF, Kurts C, Allison J, Kosaka H, Carbone F (1998). Induction of peripheral CD8+ T-cell tolerance by cross-presentation of self antigens.. Immunol Rev.

[29] Probst HC, McCoy K, Okazaki T, Honjo T, van den Broek M (2005). Resting dendritic cells induce peripheral CD8+ T cell tolerance through PD-1 and CTLA-4.. Nat Immunol.

[30] Fife BT, Bluestone JA (2008). Control of peripheral T-cell tolerance and autoimmunity via the CTLA-4 and PD-1 pathways.. Immunol Rev.

[31] Francisco LM, Sage PT, Sharpe AH (2010). The PD-1 pathway in tolerance and autoimmunity.. Immunol Rev.

[32] Parish IA, Rao S, Smyth GK, Juelich T, Denyer GS (2009). The molecular signature of CD8+ T cells undergoing deletional tolerance.. Blood.

[33] Haverkamp JM, Charbonneau B, Crist SA, Meyerholz DK, Cohen MB (2011). An inducible model of abacterial prostatitis induces antigen specific inflammatory and proliferative changes in the murine prostate.. Prostate.

[34] Kurts C, Kosaka H, Carbone FR, Miller JF, Heath WR (1997). Class I-restricted cross-presentation of exogenous self-antigens leads to deletion of autoreactive CD8(+) T cells.. J Exp Med.

[35] Shibaki A, Sato A, Vogel JC, Miyagawa F, Katz SI (2004). Induction of GVHD-like skin disease by passively transferred CD8(+) T-cell receptor transgenic T cells into keratin 14-ovalbumin transgenic mice.. J Invest Dermatol.

[36] Wang L, Dobrzynski E, Schlachterman A, Cao O, Herzog RW (2005). Systemic protein delivery by muscle-gene transfer is limited by a local immune response.. Blood.

[37] Rowe HM, Lopes L, Ikeda Y, Bailey R, Barde I (2006). Immunization with a lentiviral vector stimulates both CD4 and CD8 T cell responses to an ovalbumin transgene.. Mol Ther.

